# Auxin protects *Arabidopsis thaliana* cell suspension cultures from programmed cell death induced by the cellulose biosynthesis inhibitors thaxtomin A and isoxaben

**DOI:** 10.1186/s12870-019-2130-2

**Published:** 2019-11-21

**Authors:** Fatima Awwad, Guillaume Bertrand, Michel Grandbois, Nathalie Beaudoin

**Affiliations:** 1 Centre SÈVE, Département de biologie, Université de Sherbrooke, Sherbrooke, Québec J1K 2R1 Canada; 2 Present address: Groupe de Recherche en Biologie végétale, Département de chimie, biochimie et physique, Université du Québec à Trois-Rivières, Trois-Rivières, Québec G9A 5H7 Canada; 3 Institut de Pharmacologie de Sherbrooke, Département de pharmacologie et physiologie, Université de Sherbrooke, Sherbrooke, Québec J1H 5N4 Canada

**Keywords:** Auxin, Calcium, Cell wall, Isoxaben, Programmed cell death, Thaxtomin A

## Abstract

**Background:**

Thaxtomin A (TA) is a natural cellulose biosynthesis inhibitor (CBI) synthesized by the potato common scab-causing pathogen *Streptomyces scabies*. Inhibition of cellulose synthesis by TA compromises cell wall organization and integrity, leading to the induction of an atypical program of cell death (PCD). These processes may facilitate *S. scabies* entry into plant tissues. To study the mechanisms that regulate the induction of cell death in response to inhibition of cellulose synthesis, we used *Arabidopsis thaliana* cell suspension cultures treated with two structurally different CBIs, TA and the herbicide isoxaben (IXB).

**Results:**

The induction of cell death by TA and IXB was abrogated following pretreatment with the synthetic auxin 2,4-dichlorophenoxyacetic acid (2,4-D) and the natural auxin indole-3-acetic acid (IAA). The addition of auxin efflux inhibitors also inhibited the CBI-mediated induction of PCD. This effect may be due to intracellular accumulation of auxin. Auxin has a wide range of effects in plant cells, including a role in the control of cell wall composition and rigidity to facilitate cell elongation. Using Atomic Force Microscopy (AFM)-based force spectroscopy, we found that inhibition of cellulose synthesis by TA and IXB in suspension-cultured cells decreased cell wall stiffness to a level slightly different than that caused by auxin. However, the cell wall stiffness in cells pretreated with auxin prior to CBI treatment was equivalent to that of cells treated with auxin only.

**Conclusions:**

Addition of auxin to *Arabidopsis* cell suspension cultures prevented the TA- and IXB-mediated induction of cell death. Cell survival was also stimulated by inhibition of polar auxin transport during CBI-treatment. Inhibition of cellulose synthesis perturbed cell wall mechanical properties of *Arabidopsis* cells. Auxin treatment alone or with CBI also decreased cell wall stiffness, showing that the mechanical properties of the cell wall perturbed by CBIs were not restored by auxin. However, since auxin’s effects on the cell wall stiffness apparently overrode those induced by CBIs, we suggest that auxin may limit the impact of CBIs by restoring its own transport and/or by stabilizing the plasma membrane - cell wall - cytoskeleton continuum.

## Background

The plant cell wall plays essential roles in plant growth and development and determines cell form and size by regulating processes such as cell elongation, adhesion and water movement. It also participates in intercellular communication and offers cellular protection against biotic and abiotic aggression [1, 2]. The primary cell wall is a strong but dynamic structure that can be modified by enzymatic and non-enzymatic proteins to modulate its elasticity and extensibility in response to growth signals or environmental changes [3, 4].

Cellulose is the main structural constituent of the plant cell wall. This polysaccharide is made up of chains of β-1-4 glucose subunits linked to each other by hydrogen bonds to form microfibrils, which are embedded in a gel-like matrix comprising pectins, hemicellulose and protein [3, 5]. Cellulose microfibrils are the strongest component of the cell wall [6]. One of the most recent conceptual models of the plant cell wall organization proposes that microfibrils form bundles by direct contacts between cellulose microfibrils and at load-bearing junctions where microfibrils intertwine with xyloglucan [4]. These interactions are necessary to increase cell wall mechanical resistance. Pectins, which bind the hydrophilic surface of cellulose, fill the space between microfibrils to regulate cell wall elasticity [4, 7]. Pectins have also been implicated in maintaining and sensing cell wall integrity during salt stress [8] and pathogen interactions [9, 10].

Cellulose biosynthesis is mediated by different cellulose synthase (CESA) proteins assembled in multiprotein complexes called cellulose synthesis complexes (CSCs), which can be seen as a rosette structure in the plasma membrane [2, 5, 6, 11]. Pre-assembled CSCs are transported to the plasma membrane through the Golgi apparatus, the trans-Golgi network and ultimately into small compartments that associate with microtubules [5, 6]. Disruption of cellulose biosynthesis as induced by cellulose biosynthesis inhibitors (CBIs) perturbs the function and organization of the cell wall, altering cell expansion and cell wall integrity [6].

Several plant pathogens can perturb cell wall synthesis or organization in order to colonize plant tissues. In particular, the potato common scab pathogen *Streptomyces scabies* (syn*. scabiei*) synthesizes during the infection process a phytotoxin called thaxtomin A (TA), which is a natural cellulose biosynthesis inhibitor (CBI) [12–16]*.* TA is the main pathogenicity determinant responsible for common scab symptoms, as treatment of potato tubers with TA induces scab-like symptoms [17–19] and inhibition of TA synthesis in normally pathogenic strains abolishes the formation of scab-like symptoms on infected tubers [20, 21]. It was proposed that the actinobacterium *S. scabies* would use TA to facilitate bacterial penetration of plant cell walls [15]. However, the specific action of TA on the cell wall organization and integrity is not known yet.

At the plant level, the effects of TA are very similar to those induced by the well-known CBI isoxaben (IXB). In *Arabidopsis thaliana* seedlings, TA causes a reduction of growth, root swelling, induction of ectopic lignification and defense-related gene expression [16, 22–26]. While IXB specifically targets CESA3 and CESA6 [27, 28], the specific molecular target of TA is unknown. However, it is most probably different from that of IXB, as mutants resistant to IXB are not resistant to TA [29]. Moreover, TA induces a pattern of ectopic lignification different than that induced by IXB, and changes in gene expression induced by TA are not entirely mimicked by IXB treatment [22, 26].

In *Arabidopsis* cell suspensions, inhibition of cellulose biosynthesis by IXB or TA triggers cellular hypertrophy and induces an atypical program of cell death (PCD) [30]. However, little is known on the regulation of this unusual PCD. It was shown that TA-induced PCD depends on a calcium influx and is abrogated by transcription and translation inhibitors [30, 31]. TA-induced PCD is not associated with H_2_O_2_ production and does not involve defense-related mitogen-activated protein kinase (MAPK) signaling [26, 30, 31]. Since TA is the main pathogenicity factor of potato common scab, reducing the impact of TA on plant cells stands out as a promising strategy to counter this disease. It was reported that resistance to common scab was increased by spraying potato plants with the synthetic auxin 2,4-dichlorophenoxyacetic acid (2,4-D) at tuber induction, an effect that was attributed to decreased TA toxicity in tubers [32]. Similarly, TA inhibition of *Arabidopsis* seedling growth can be reversed by the synthetic auxin 2,4-D or the natural auxin indole-3-acetic acid (IAA) [32]. However, it is not clear how auxin treatment can reduce TA toxicity in tubers or seedlings.

In this work, we used *Arabidopsis* cell suspensions to study the mechanisms that regulate the induction of cell death in response to TA at the cellular level. We showed that synthetic and natural auxin pretreatment can protect cells from TA-induced cell death. This protection was not specific to the TA molecule itself; auxin pretreatment also abrogated cell death induced by IXB. Moreover, inhibitors of calcium influx and auxin efflux inhibitors decreased the induction of cell death by both CBIs. Auxin induces changes in cell wall composition and increases cell wall loosening, which facilitates cell elongation. Using AFM in force measurement mode, we found that TA or IXB treatment led to diminishing cell wall stiffness at a level significantly different than that observed after auxin treatment. However, the combination of auxin pretreatment followed by CBI addition reduced cell wall stiffness to a level equivalent to that observed after auxin treatment alone. We discuss how auxin may restrain the impact of inhibition of cellulose synthesis by stimulating rapid changes in the plasma membrane - cell wall interface.

## Results

### Increase in cytosolic calcium is necessary for CBI-induced cell death

**Fig. 1:**
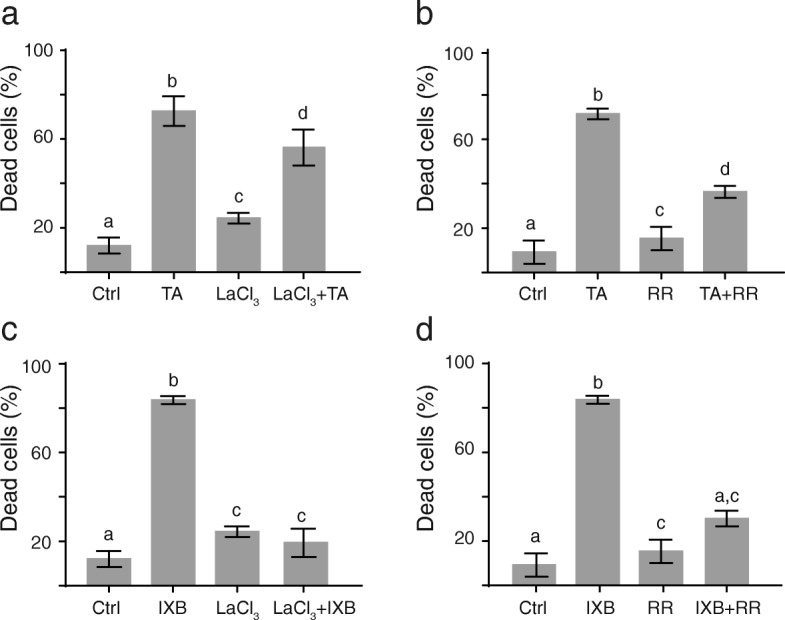
Calcium influx is required for the CBI-induction of cell death. Percentage of dead cells in *Arabidopsis* suspension cells 48 h after the addition of calcium transport inhibitors. **a** and **c** Cells were treated with thaxtomin A (TA; 1 μM), isoxaben (IXB; 1 μM) or lanthanum chloride (LaCl_3_; 500 μM) alone, or pretreated with LaCl_3_ (500 μM) 30 min before adding TA or IXB. **b** and **d** Cells were treated with TA (1 μM), IXB (1 μM) or ruthenium red (RR; 50 nM) alone, or pretreated with 50 nM of RR 30 min before adding TA or IXB. Ctrl = control cells treated with equal volume of methanol as treated samples. Each value is the mean of *n* = 15 (100 cells each) ± SD. Statistically different values (t-test followed by Holm-Šídák method, *p* < 0.05) are indicated by different letters

A rapid and short calcium influx has been measured in *Arabidopsis* cells and seedlings in response to TA treatment [31, 33]. Pretreatment of cell cultures with the calcium channel inhibitor lanthanum chloride (LaCl_3_) inhibited TA-induced cell death, indicating that the increase in cytosolic calcium was required to activate the signaling cascade leading to the induction cell death by TA in *Arabidopsis* cells [31]. The inhibitor of cellulose synthesis IXB also activated a program of cell death very similar to that induced by TA, but the implication of calcium in IXB-induced cell death had never been investigated. To determine whether calcium influx was required for the IXB-induced cell death, we used different calcium transport inhibitors (Fig. 1); LaCl_3,_ which inhibits extracellular calcium uptake, and ruthenium red (RR), which putatively blocks calcium release from intracellular stores and inhibits the mitochondrial calcium uniporter [34–36]. These inhibitors were added to *Arabidopsis* cell cultures 30 min before adding TA or IXB. As reported before [31], LaCl_3_ significantly reduced the percentage of cell death in TA-treated cells from 72% down to 56% (Fig. 1a). The effect of LaCl_3_ was even more important in IXB-treated cells, with a reduction of cell death from 84% down to 19% (Fig. 1c). Pretreatment with RR also inhibited the induction of cell death mediated by both CBIs, decreasing cell death by 36% in TA-treated cells and by 54% for IXB treatment (Fig. 1b and d). Both treatments also inhibited the typical cell bulging observed in response to CBI [30] and decreased the average cell size (Table 1; Additional file 1), indicating that these inhibitors may affect cell elongation.

**Table 1:** Distribution of cells according to cell length (μm) 48 h after treatment with cellulose biosynthesis inhibitors, inhibitors of calcium transport and auxins

Treatment	Percentage of cells according to cell length^a^		
	10–40 μm	40–60 μm	> 60 μm
Control (Methanol)	44.0 ± 11.7	33.4 ± 2.4	26.5 ± 9.3
Thaxtomin A	75.5 ± 2.2*	22.6 ± 0.2	4.1 ± 0.2*
Isoxaben	67.9 ± 4.7*	27.1 ± 1.5	10.9 ± 6.2*
LaCl_3_	92.6 ± 4.3*	4.7 ± 1.8*	2.7 ± 0.6*
RR	85.8 ± 5.6*	12.8 ± 2.7*	1.5 ± 1.0*
2,4-D	89.6 ± 6.3*	7.2 ± 3.3*	3.2 ± 2.8*
IAA	72.1 ± 8.6*	17.5 ± 5.7*	10.4 ± 4.6*

^a^Cells stained with trypan blue were visualized by light microscopy and photographed. Cell dimensions were measured using Fiji software [37] for 300 cells per condition. Results show the mean percentage of cells in each category of length from 3 replicates ± SD. (*) indicates statistically significant difference with control in each category (t-test followed by Holm-Šídák method, *p* < 0.05)

### Auxin protects *Arabidopsis* cells from CBI-induced cell death

**Fig. 2:**
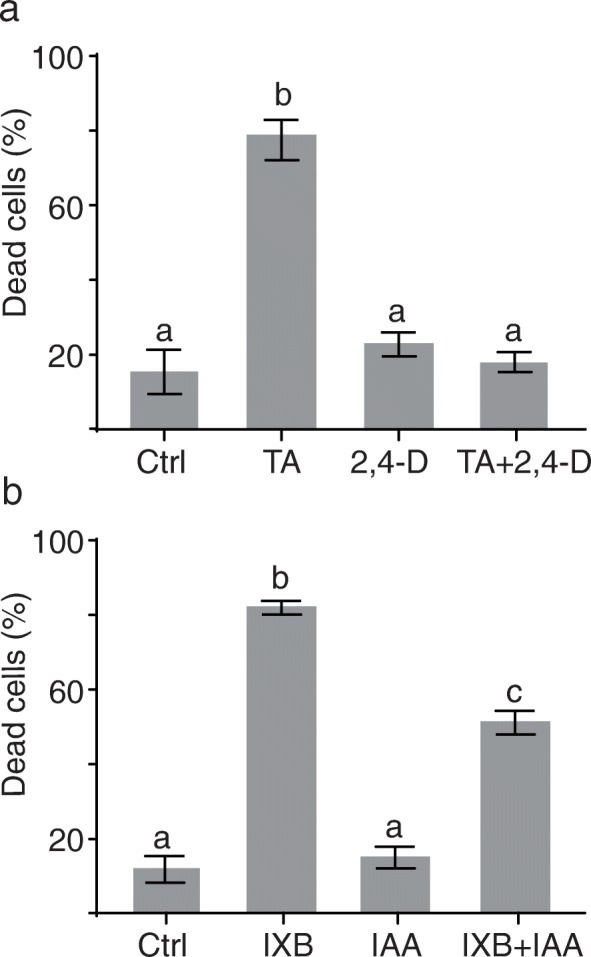
Auxin protects *Arabidopsis* cells from CBI-induced cell death. Percentage of dead cells in *Arabidopsis* suspension-cultured cells 48 h after addition of auxin and/or CBI. **a** Cells were treated with thaxtomin A (TA; 1 μM), 2,4-dichlorophenoxyacetic acid (2,4-D; 50 μM), or 2,4-D 30 min before adding TA. **b** Cells were treated with isoxaben (IXB; 1 μM), IAA (1 μM), or IAA (1 μM) 30 min before adding IXB. Ctrl = control cells treated with equal volume of methanol as treated samples. Each value is the mean of *n* = 15 (100 cells each) ± SD. Statistically different values (t-test followed by Holm-Šídák method, *p* < 0.05) are indicated by different letters

Since 2,4-D has been proposed to reduce TA toxicity in *Arabidopsis* seedlings and potato tubers [32], we determined whether it could also protect from TA-induced cell death at the cellular level. *Arabidopsis* cell suspension cultures were pretreated for 30 min with the synthetic auxin 2,4-D prior to the induction of cell death with TA. Dead cells were counted periodically over a period of 72 h. As shown in Fig. 2a, the percentage of dead cells 48 h after TA addition was 79% whereas cultures pretreated with 2,4-D before adding TA showed only 18% of dead cells (Fig. 2b). This significant inhibition of cell death may be specifically attributed to 2,4-D or may be a more general response to auxin. To answer this question, we pretreated cells with the natural auxin IAA or the synthetic auxin 1-naphtalenacetic acid (NAA) before adding TA. As shown in Additional file 2, IAA pretreatment alone increased the percentage of cell death to 48% within 48 h, but it significantly decreased the percentage of cell death induced by TA, with 49% of cell death in IAA-pretreated cells compared to 79% in TA only-treated cells. However, pretreatment of cell cultures with NAA at low concentration (1 and 10 μM) was not effective in protecting from cell death induced by TA. When we used higher concentrations (30 μM; Additional file 2), NAA-pretreatment alone increased cell death at a level similar to that induced by TA, making it impossible to determine whether NAA could effectively alter the response to TA.

Auxins may exert their protective effect directly against the TA molecule itself (e.g., competition, interaction) or may protect cells from TA’s ability to inhibit cellulose synthesis or its downstream consequences. To discriminate between these possibilities, we repeated the previous experiments using a structurally different inhibitor of cellulose biosynthesis IXB, which also induced PCD in *Arabidopsis* cell cultures [30]. *Arabidopsis* cell suspension cultures were pretreated for 30 min with various concentrations of 2,4-D, IAA or NAA followed by IXB treatment. All auxin pretreatments at a concentration of 1 μM were able to reduce significantly the percentage of cell death induced by IXB, but this reduction was of only 11% in 2,4-D pretreated cells (Additional file 3). The protective effect of the natural auxin IAA against IXB is presented at Fig. 2b. Pretreatment with IAA prior to IXB addition reduced the percentage of cell death from 84% in IXB-treated cells down to 52% in IAA-pretreated cells 48 h after the addition of IXB.

### Inhibition of auxin efflux protects cells from CBI-induced cell death

Decreased cellulose synthesis by TA and IXB significantly reduced cell elongation within 48 h in *Arabidopsis* cell suspension cultures (Table 1). However, CBI induced a radial expansion (hypertrophy and bulging) of cells at the end of cell files in suspension-cultured cells ([30]; Additional file 1). Interestingly, hypertrophied cells remained alive longer than other cells in response to TA or IXB. This observation suggests the polarized accumulation of a compound or signal that would stimulate cell survival at the end of cell files. Auxin stands out as a good candidate as this hormone is transported in a directional and polarized fashion in plants as well as in tobacco BY-2 cell suspension cultures [35]. In light of our results with auxins, we hypothesized that the enhanced survival of hypertrophied cells may be associated with the polar transport and accumulation of auxin at the end of cell files. Accordingly, addition of exogenous auxin to cell cultures would increase the intracellular level of auxin and thus promote cell survival in most cells even after CBI-treatment.

**Fig. 3:**
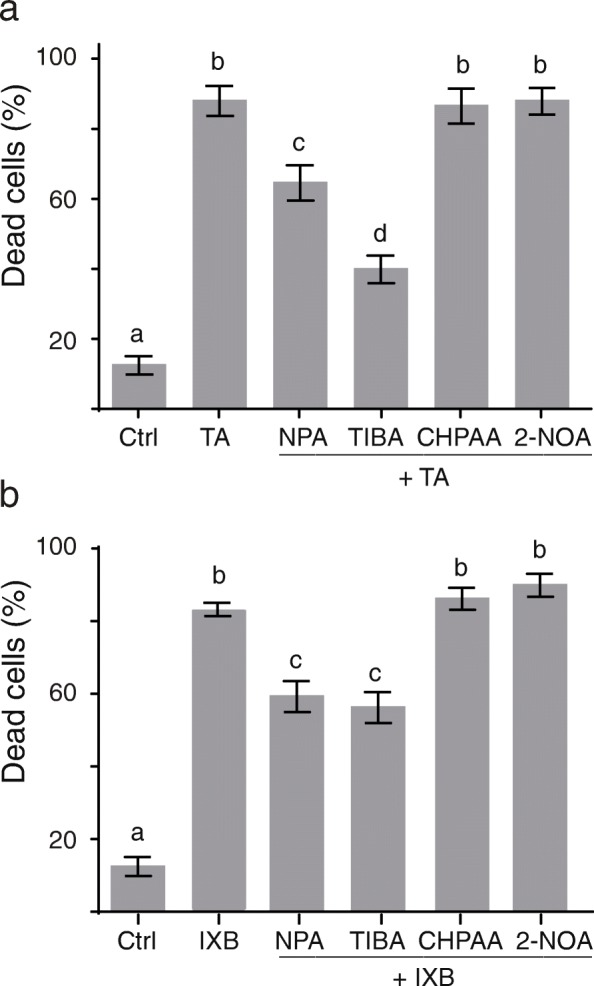
Inhibition of auxin transport (efflux) protects from CBI-induced cell death. Percentage of dead cells in *Arabidopsis* cell cultures 72 h after addition of auxin transport inhibitor and/or CBI. **a** Cell death was evaluated after treatment with thaxtomin A (TA; 1 μM) and in cells pretreated for 30 min with 10 μM of different auxin transport inhibitors: 2,3,5-triiodobenzoic acid (TIBA) and 1-N-naphthylphthalamic acid (NPA) as efflux inhibitors, 2-naphthoxyacetic acid (NOA) and 3-chloro-4-hydroxyphenylacetic acid (CHPAA) as influx inhibitors prior to TA treatment. **b** Cell death was evaluated after treatment with isoxaben (IXB; 1 μM) and in cells pretreated for 30 min with the different auxin transport inhibitors prior to IXB addition. Ctrl = control cells treated with equal volume of methanol as treated samples. Each value is the mean of *n* = 15 (100 cells each) ± SD. Statistically different values (t-test followed by Holm-Šídák method, *p* < 0.05) are indicated by different letters

To evaluate the importance of polar auxin transport in the induction of cell death by CBI, we treated cell suspensions with two different auxin efflux inhibitors, i.e., *N*-1-naphthylphthalamic acid (NPA) and triiodobenzoic acid (TIBA), and two auxin influx inhibitors, i.e., 3-chloro-4-hydroxyphenylacetic acid (CHPAA) and 2-naphthoxyacetic acid (2-NOA). Since NPA and TIBA also have other effects in plant cells, including perturbation of vesicle trafficking and cytoskeleton, we used a concentration for each chemical that did not alter the cytoskeleton [38–40]. NPA or TIBA was added to *Arabidopsis* cell suspensions 30 min prior to CBI-treatment. Dead cells were counted over a period of 48 h. As shown in Fig. 3a, the percentage of dead cells in TA-treated cells was significantly decreased by pretreatement with NPA and TIBA from 87% down to 65 and 40% respectively. In contrast, pretreatment with auxin influx inhibitors, such CHPAA and 2-NOA, had no effect on TA-induced cell death. Again, this reduction in cell death was not specific to the TA molecule itself as it was observed in IXB-treated cell cultures as well. Cell cultures pretreated with NPA or TIBA before the addition of IXB had fewer dead cells (25 to 28%) than IXB-treated cell cultures (Fig. 3b). This data shows that blocking auxin efflux using TIBA and NPA increases the survival rate of CBI-treated cells.

### CBI and auxin decrease cell wall stiffness and restrain cell elongation

Inhibition of cellulose synthesis by TA and IXB in *Arabidopsis* cell cultures and seedlings induced the expression of genes involved in cell wall synthesis and ectopic accumulation of lignin [22, 26]. These responses were suggested to play a role in the reinforcement of the cell wall compromised by impaired cellulose synthesis. This could maintain cell wall mechanical properties and integrity required for cell survival, elongation and growth [22]. Auxin is also important for controlling cell wall composition and can induce cell wall loosening that is required for cell elongation. Since both CBI and auxin have an impact on the cell wall, it is possible that the protective effect of auxin against CBI is associated with their selective effects on the mechanical properties of the cell wall.

**Fig. 4:**
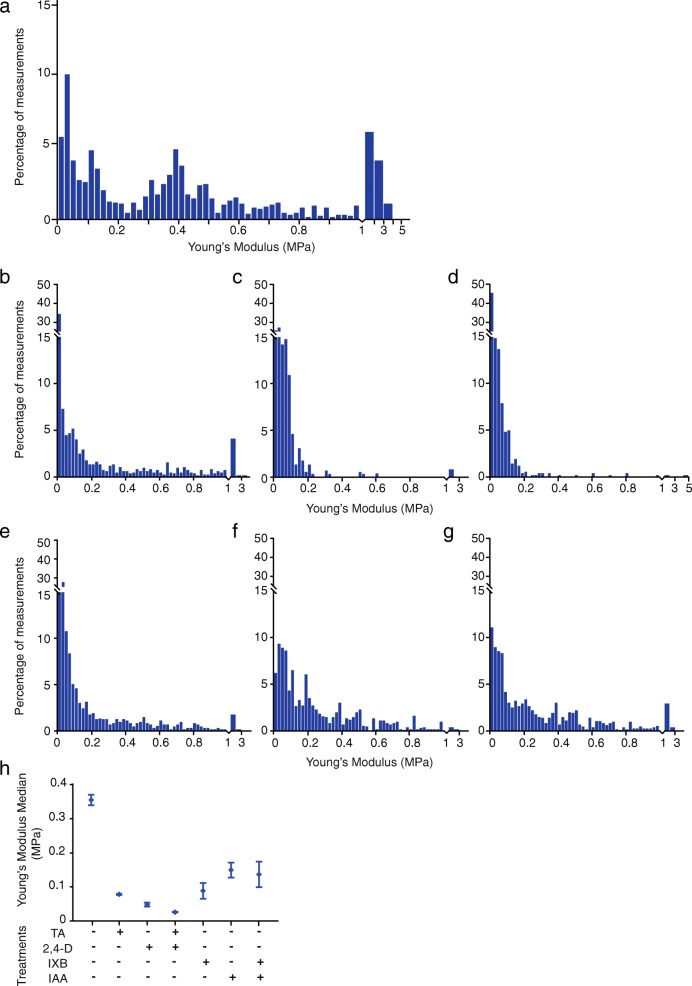
CBIs and auxin decrease cell wall stiffness. Atomic force microscopy (AFM) was used to measure cell wall stiffness profiles of *Arabidopsis* suspension-cultured cells treated with: **a** methanol (control), **b** thaxtomin A (TA; 1 μM), **c** 2,4-dichlorophenoxyacetic acid (2,4-D; 50 μM), **d** 2,4-D and TA, **e** isoxaben (IXB; 1 μM), **f** IAA (1 μM), **g** IAA and IXB for 24 h. Force curves were recorded at the surface of 3 cells per condition in order to extract Young’s modulus (MPa) values (see Material and Methods). Each histogram bar represents the relative proportion of Young’s modulus values measured on the cell surface in each condition. Because of the low proportion of values measured above 1 MPa, a scale change was used after 1 MPa. **h** Average median values of Young’s modulus were calculated for each condition from 3 different experiments (3 to 4 cells per experiment). Error bars represent SD

To determine the impact of TA and IXB as well as auxin on cell wall mechanical stiffness, we used Atomic Force Microscopy (AFM)-based force spectroscopy to measure mechanical properties expressed as the elastic modulus of the cell wall in living cells, as described previously [41]. Here, this technique allows a non-destructive analysis of cells in order to assess the impact of CBI on the mechanical properties of the cell wall in living cells undergoing PCD. Young’s modulus was extracted from force indentation curve recorded on cells treated for 24 h with each CBI, auxin or a combination of both (Fig. 4). We chose to perform AFM measurements on non plasmolysed cells in liquid medium as plasmolysis may change the cell response to CBI or auxin. In particular, plasmolysis can recapitulate some of the effects of IXB treatment [42, 43]. Plasmolysis and IXB modify the plasma membrane localization of PIN auxin efflux transporters, which can alter polar auxin transport [42]. Plasmolysis can also perturb the plasma membrane - cell wall - actin cytoskeleton continuum, as it was shown to alter actin remodelling and Golgi body motility [43]. Finally, we found that plasmolysis treatment slightly reduced CBI-induced PCD (Additional file 4), indicating that perturbation of the plasma membrane-cell wall interface by plasmolysis can alter the induction of PCD by CBI, the object of this study.

*Arabidopsis* cells treated with the indicated concentrations of auxin and CBI were compared to control cells originating from the same culture. Hence, any differences in the elastic modulus (Young’s modulus) measured on the cell surface could be attributed to changes induced by the different CBI and auxin treatments. The histograms presented at Fig. 4 shows the distribution percentage of measured Young’s modulus with values ranging from 0 to 5 MPa for each treatment. We also calculated the average median of Young’s modulus values for each treatment as summarized in Fig. 4h.

In control cells (Fig. 4a), Young’s modulus values were distributed over a broad range, with close to 39% of values found below 0.2 MPa, and 50% found between 0.2 and 1 MPa. The average Young’s modulus median value of cell walls in control cells was 0.355 MPa, which is comparable to results also obtained in *Arabidopsis* suspension cells by Radotić et al. (2012). In comparison to control cells, we observed a steep decrease in cell wall stiffness in cells treated with TA and IXB (Fig. 4b and e) with more than 67 and 68% respectively of the measurements below 0.2 MPa. Accordingly, the average Young’s modulus median values (Fig. 4h) calculated for cell walls of TA- or IXB-treated cells, i.e., 0.078 and 0.089 MPa respectively, were lower than the Young’s modulus average median obtained in control cells (0.355 MPa), demonstrating a significant drop in cell wall stiffness. This indicates that inhibition of cellulose synthesis drastically perturbed the mechanical properties of the cell wall.

Auxin’s effect on cell wall mechanical properties was also evaluated. In 2,4-D-treated cells (Fig. 4c), more than 83% Young’s modulus values were found below 0.1 MPa, with close to 94% found below 0.2 MPa, indicating a drastic reduction in cell wall stiffness compared to control cells. The combination of both 2,4-D and TA treatments decreased cell wall stiffness to a level comparable to that caused by 2,4-D (Fig. 4d), with close to 87% Young’s modulus values found below 0.1 MPa and up to 96% found below 0.2 MPa. As shown in Fig. 4h, the average Young’s modulus median of cell walls from cells treated with 2,4-D alone (0.049 MPa) or in combination with TA (0.026 MPa) was lower than the average Young’s modulus median value of TA-treated cells (0.078 MPa). While the combined 2,4-D and TA treatments may contribute to the enhanced reduction in cell wall stiffness observed in comparison to cells treated with TA only, the distribution of Young’s modulus values obtained after 2,4-D and TA combination closely resembled that of 2,4-D treated cells, suggesting that the effect of 2,4-D somehow superseded that of TA.

Cells treated with the natural auxin IAA alone also showed a decreased cell wall rigidity when compared to control cells, with 37% Young’s modulus values distributed below 0.1 MPa and 58% below 0.2 MPa (Fig. 4f). The average Young’s modulus median was of 0.149 MPa (Fig. 4h). When IAA and IXB treatments were combined (Fig. 4g), the Young’s modulus distribution of values was comparable to that observed after IAA treatment alone, with close to 40% Young’s modulus values below 0.1 MPa and close to 52% below 0.2 MPa. In this case, average Young’s modulus median value of the combined treatment was 0.137 MPa (Fig. 4h), which is higher than the average Young’s modulus median value (0.089 MPa) obtained in cells treated with IXB only, suggesting that the reduction in cell wall stiffness induced by IXB was at least partially overridden by the effect of IAA.

Despite this drastic decrease in cell wall rigidity, we observed that *Arabidopsis* cell cultures treated with auxin or with CBIs exhibited a higher proportion of cells smaller than control cells (Table 1). In response to a 48 h-treatment with TA or IXB, 76 and 68% of cells respectively remained shorter than 40 μm compared to 44% in control cells. This proportion was similar in IAA-treated cell cultures, with 72% of cells in the 10–40 μm category. Treatment with 2,4-D reduced cell elongation even more than CBIs or IAA, with over 89% cells in the 10 to 40 μm category. Moreover, only 4 to 10% of TA- or IXB-treated cells and 3 to 10% in 2,4-D or IAA-treated cells were longer that 60 μm compared to 26% in control cells. These results show that both CBIs and auxin decreased cell elongation in these experimental conditions.

## Discussion

Inhibition of cellulose biosynthesis by TA and IXB in *Arabidopsis* cell cultures induced an atypical programmed cell death [30]. In most cases, defense-related PCD is preceded by a calcium influx that is associated with an oxidative burst characterized by the production of H_2_O_2_ [44] and the activation of a MAPK signaling pathway [45, 46]. However, TA did not induce the production of H_2_O_2_ nor activated defense-associated MAPK pathways [30, 31], demonstrating that the induced PCD was different from the classical defense-related PCD. However, in *Arabidopsis* cells and seedlings, TA induced a rapid and short calcium influx essential for the induction of cell death and inhibition of growth [31, 33]. Rapid accumulation of cytosolic calcium can be detected in response to plant pathogens, specific elicitors and toxins but also in response to mechanical stimuli [47–49]. As reported for TA-induced PCD [31], the induction of cell death by IXB was inhibited by pharmacological inhibitors that block calcium influx (LaCl_3_) and calcium release from internal stores (RR) (Fig. 1). These results provide evidence that changes in cytosolic calcium, which occurred after inhibition of cellulose synthesis, are early steps in the signalling cascade that leads to PCD, as observed in several types of PCDs [50].

TA is a natural biosynthesis inhibitor and the main pathogenicity factor of *Streptomyces scabies*, a filamentous actinobacterium causing the potato tuber disease common scab [17, 18]. TA inhibits root growth in *Arabidopsis*, tomato and potato plants [33] and perturbs *Arabidopsis* seedling growth, an effect that can be reversed by the synthetic auxin 2,4-D or the natural auxin IAA [32]. In this work, we investigated the effect of auxin on TA-induced cell death at the cellular level using *Arabidopsis* cell suspension cultures. Pretreatment of *Arabidopsis* cell suspension cultures with 2,4-D or with the natural auxin IAA significantly increased cell survival of TA-treated cells when compared to control cells (Fig. 2a; Additional file 2). The synthetic auxin 2,4-D was more efficient than IAA at protecting from TA-induced cell death. This may be explained in part by the faster metabolic degradation of IAA compared to the metabolically stable 2,4-D [51]. These results demonstrate that both the natural auxin IAA and the synthetic auxin 2,4-D can significantly protect plant cells from the effects of TA. Synthetic and natural auxins were also very efficient in inhibiting cell death in response to IXB, another CBI structurally different than TA (Fig. 2b; Additional file 3). In this case, even low levels (1 μM) of the various auxins were able to significantly reduce cell death. Here, the most efficient auxin was NAA while 2,4-D was the least efficient.

Overall, these results show that auxin protects cells against the consequences of inhibition of cellulose synthesis and not directly against the TA or IXB molecules. However, the level of protection against cell death varied depending on which auxinic compound and CBI was used. These variations may be due to differences in uptake, activity or rates of metabolic degradation for each auxinic molecule [51] or to the different modes of action of TA and IXB.

Pretreatment of *Arabidopsis* cell cultures with the auxin efflux transport inhibitors NPA and TIBA also increased cell survival in cells treated with CBI (Fig. 3). In contrast, auxin influx inhibitors CHPAA and 2-NOA had no not effect on CBI-induced cell death (Fig. 3). There are several examples which showed that blocking auxin efflux using NPA and TIBA can increase the accumulation of auxin in plant cells and plant tissues [35, 38, 51–56]. For instance, tobacco cells and *Arabidopsis* suspension cultures grown in the presence of radiolabelled auxins and the auxin efflux inhibitors TIBA and NPA had an increased accumulation of radiolabeled auxin [38, 51–53, 55]. The synchronization of cell division in tobacco cells, a process which depends on polar auxin transport, was also perturbed by NPA; this effect was attributed to increasing intracellular concentration of auxin due to reduced auxin efflux [35, 53]. In another experiment using citrus explants expressing an auxin-inducible *GUS* reporter gene, it was shown that polarized accumulation of auxin at the basal end of the stem cut was perturbed by NPA treatment, which induced an even distribution of auxin across the explant [56]. Finally, blocking auxin efflux with NPA in bean leaf petiole significantly enhanced accumulation of free IAA in leaf blades [54]. Based on these results, we suggest that inhibition of CBI-induced cell death by auxin efflux inhibitors is most likely due to the accumulation of endogenous auxin in cells. However, blocking auxin transport can also perturb auxin homeostasis within plants cells. To control intracellular auxin levels, various cellular mechanisms may be activated, including reduced auxin synthesis and conjugation or oxidation to inactive forms [57]. Whether these mechanisms are involved in the inhibition of CBI-induced cell death has not been investigated.

Other reports have also shown that auxin accumulation can inhibit PCD occurring during plant development or in response to stress. For example, the development of Norway spruce (*Picea abies*) somatic embryos involves the programmed elimination of suspensor and embryonal tube cells by PCD [58]. Inhibition of auxin transport using NPA inhibited PCD in suspensor and tube cells, suggesting that auxin accumulation in these cells perturbed PCD and embryo patterning [58]. Auxin has also been involved in the protection of stem cell niche from chilling stress-induced PCD in root tissues [59]. In normal conditions, auxin concentration in root stem cell niche is maximal in the quiescent center (QC) and follows a local gradient at the root tip. This auxin distribution allows division of root stem cells and inhibits division of columella stem cell daughters (CSCD), which are committed to differentiate. Chilling stress causes DNA damage that specifically induces PCD in CSCD whereas other cells from the root stem cell niche survive. Chilling stress also perturbs the distribution of auxin; low auxin levels contribute to PCD of CSCD, which in turn re-establish an auxin maximum in the stem cell niche to protect root stem cells from division and DNA-damage induced by chilling stress [59]. These examples and our results show that auxin plays a role in promoting cell survival during specific developmental stages or in response to various external conditions. However, how auxin can inhibit cell death in these cases has not yet been unravelled.

In the case of CBI-induced PCD, it is possible that auxin exerts its protective effect directly at the level of the cell wall. Auxin induces the expression of cell wall-wall related genes and stimulates trafficking of vesicles containing new cell wall material [60, 61]. Auxin can also regulate a variety of cell wall modifying enzymes including expansins and pectin methylesterase to control cell elongation [61, 62]. Regulation of cell elongation by auxin involves changes in the mechanical properties of the cell wall. This effect is explained by the “acid growth theory” which stipulates that extracellular acidification caused by auxin induces cell wall loosening to increase cell wall extensibility and cell expansion [61]. Auxin can also reduce cell wall stiffness through demethylesterification of the pectin homogalacturonan as observed in the shoot apex of *Arabidopsis thaliana* prior to organ outgrowth [63]. Hence, it is possible that auxin-mediated changes in the mechanical properties of the cell wall limit the impact of CBI.

We used AFM-based force spectroscopy to evaluate the effect of CBI and auxin on the mechanical properties of the cell wall of *Arabidopsis* suspension-cultured cells. This technique is non-destructive and can be used in living cells. The mechanical properties of the cell wall were expressed as the elastic modulus (Young’s modulus) as described before [41]. As shown in Fig. 4, the average Young’s modulus median value of the cell wall of control cells was around 0.355 MPa while this value was down to 0.078 and 0.089 MPa in TA and IXB-treated cells respectively. This showed that CBI caused a drastic decrease in cell wall stiffness. This also demonstrated that the induced expression of cell wall genes in response to TA and IXB [22, 26] was not sufficient to compensate for changes caused by CBI. Similarly, auxin treatment also reduced cell wall stiffness; cells treated with 2,4-D or IAA had an average Young’s modulus median values of 0.049 and 0.149 MPa respectively. Interestingly, the pattern of distribution of Young’s modulus values of cells treated with a combination of auxin and CBI was very similar to that of cells treated with auxin alone (Fig. 4). Changes induced by treatment with 2,4-D seemed to overcome those induced by TA (Fig. 4b-d). However, reduction in stiffness caused by IXB was partially overcome by pretreating cells with IAA.

Overall, these results indicate that CBI and auxin treatments all reduce cell wall stiffness compared to control cells. Moreover, this data shows that auxin treatment does not enhance cell survival by restoring the cell wall rigidity in CBI-treated cells. On the other side, the AFM data also suggests that the impacts of auxin treatment on the cell wall rigidity superseded at least partially those induced by CBI.

Increased cell wall extensibility by auxin is generally required to stimulate rapid cell elongation. However, we observed that *Arabidopsis* cell cultures treated with auxin contained a high proportion of cells smaller than control cells (Table 1), indicating that cell elongation was reduced by auxin despite the decreased cell wall stiffness. While auxin generally stimulates cell elongation, high auxin concentrations are inhibitory in root tissues [60, 64]. It is possible that results obtained in dark-grown suspension cell cultures may partly mimic the effect observed in root tissues. Recently, it was shown that increasing endogenous or exogenous levels of auxin in root tissues induced a transient apoplast alkalinisation that would be responsible for inhibiting root elongation [65]. As observed in roots, addition of exogenous auxin reduced cell elongation in *Arabidopsis* suspension cultured-cells (Table 1). Similarly, inhibition of cellulose synthesis by TA and IXB also decreased cell elongation in *Arabidopsis* cells (Table 1) and was previously shown to impair root growth in seedlings [22, 25, 33]. Interestingly, a biphasic change in pH was measured after TA treatment of *Arabidopsis* cell suspensions, with a short acidification period that was followed by a large alkalinisation [31]. It is possible that changes in pH by auxin and CBI contributed to the inhibition of cell growth observed in this study. Overall, these results demonstrate that decreased cell wall stiffness does not necessarily correlates with stimulation of cell elongation.

Our results indicate that the mode of action of auxin in promoting cell survival in CBI-treated cells is clearly not limited to changes in cell wall mechanical properties. Several other possibilities will need to be explored to fully understand how auxin protects against CBI. For instance, it was shown that induction of cell wall defects by IXB perturbed the polarized localization of auxin PIN transporters, thus altering polarized auxin transport [66]. Cellulose synthesis is governed by microtubules, which control the orientation of CSC movement directing the orientation of cellulose microfibrils. In addition, microtubules appear to control PIN polarity in a cell specific manner [67]. The PIN auxin efflux carriers are localized in membrane microdomains associated with the cellulose-synthesizing CSCs. Therefore, perturbation of cellulose synthesis may compromise microtubule stability, altering PIN localization and inhibiting cell elongation [67]. Since auxin can itself enhance expression of PIN transporters and re-establish their polarized localization [68–71], auxin treatment can potentially restore auxin transport that was perturbed by cell wall defects. Restoration of PIN localization may stabilize their association with CSCs, which could in turn partially restore cell wall integrity by resuming cell wall synthesis. This hypothesis could also explain why the auxin-mediated decrease in cell wall stiffness seemed to override that induced by CBI.

Microtubules appear to work together with actin filaments to maintain auxin fluxes and establish auxin maxima. Therefore, changes in the configuration of the actin cytoskeleton may also play a role in the auxin-mediated protection against CBI. The induction of cell wall defects by IXB can stimulate actin bundling [43] which is an early and essential event in PCD [72]. Auxin treatment can restore normal actin configuration [73, 74]. It was shown by Chang et al. (2015) that re-establishment of a normal actin configuration is involved in the auxin-mediated protection against PCD induced in tobacco leaves by the proteinaceous elicitor harpin [75]. The protective effect of auxin would rely on modifications of the level of actin organization and its interaction with the plasma membrane [75]. Hence, it is possible that auxin prevents cell death induced by CBI by restoring a normal actin configuration that in turn would stabilize the plasma membrane - cell wall - cytoskeleton continuum.

## Conclusion

We have shown that exogenous addition of synthetic and natural auxins to *Arabidopsis* cell cultures can inhibit PCD induced by two structurally different CBIs, TA and IXB. For both CBIs, initiation of cell death depended on an increase in cytosolic calcium originating from external and internal sources, which shows that calcium is involved at an early step in the signaling cascade leading to CBI-induced PCD. Addition of auxin efflux inhibitors (NPA and TIBA), which can increase the accumulation of auxin, was also shown to protect cells from CBI-induced PCD. These findings support work by others who showed that specific development- or defense-related PCDs can be inhibited by auxin. Auxin may also protect cells from CBI-induced cell death by inducing changes in the cell wall composition and organization to compensate for reduced cellulose synthesis. However, since cell wall stiffness evaluated by AFM-based force spectroscopy was found to be reduced both by CBI-inducers of PCD and auxin, we concluded that auxin-mediated protection against CBI-induced PCD does not rely on reinforcement of the cell wall. We suggest that auxin may compensate for cell wall defects by stimulating rapid changes in the plasma membrane - cell wall interface that could restore auxin transport and/or by re-establishing the plasma membrane - cell wall - cytoskeleton continuum important for cell survival. Further investigation will be required to evaluate these possibilities.

## Methods

### Plant material and treatments

*Arabidopsis thaliana* accession Landsberg *erecta* cell suspension cultures were graciously provided by Dr. Jean Rivoal (IRBV, Montréal, PQ, Canada). All chemicals were purchased from Sigma Aldrich unless otherwise indicated. Cell suspensions were grown in 45 mL Murashige and Skoog (MS) medium (pH 5.7) supplemented with B5 vitamins and 1 mg L^− 1^ 2,4-dichlorophenoxyacetic acid (2,4-D) in 125 mL Erlenmeyer flasks kept on a rotary shaker (120 rpm) at 22 °C in the dark. *Arabidopsis* cell cultures were subcultured every 7 d by diluting 15 mL cells into fresh medium. Treatments were performed using 10 mL log-phase cells 3 to 4 d after subculture. Thaxtomin A (TA) was prepared as described before [30]. TA (stock of 1 mM) and isoxaben (IXB; stock of 10 mM) were prepared in methanol and added at a final concentration of 1 μM. The same volume of methanol (less than 0.1% of final volume) was added to control cells. 2,4-D and IAA were dissolved in ethanol and 1-naphtalenacetic acid (NAA) in water. These chemicals were added at the final concentration indicated in each experiment. Auxin transport inhibitors triiodobenzoic acid (TIBA), *N*-1-naphthylphthalamic acid (NPA), 2-naphthoxyacetic acid (2-NOA) and 3-chloro-4-hydroxyphenylacetic acid (CHPAA) were dissolved in ethanol and added to cell cultures at a final concentration of 10 μM. Calcium inhibitors ruthenium red (RR) and lanthanum chloride (LaCl_3_) were diluted in water and filtered. Cells were treated with a final concentration of 50 nM of RR and 500 μM of LaCl_3_.

### Cell death assay

Cell death was assessed using trypan blue staining as described before [30]. For microscopic evaluation, cells were incubated for 5 min in a fresh medium containing fluorescein diacetate (FDA) at a final concentration of 50 μM and propidium iodide (PI) at a final concentration of 2.78 μM. Images were taken with a Zeiss Imager Z1 microscope (Carl Zeiss Canada Ltd., Ontario, Canada) equipped with a monochromatic camera using AxioVision 4.8.2 version. Forty μL of cell culture were examined for living cells (FDA fluorescence) and dead cells (PI fluorescence). FDA was visualized with excitation wavelength of 488 nm and emission was collected between 510 and 530 nm. PI was excited at 540 nm and emission was collected above 590 nm (590LP). For each condition, at least 500 cells in groups of 100 were counted. Each experiment was repeated at least three times.

### Measurement of cell dimensions

Cell dimensions were determined using Fiji software [37] by measuring the length of 300 cells per condition from three independent experiments.

### Cell surface mechanics

The elastic modulus (Young’s modulus) of individual cells was quantified from force/tip-sample separation curves recorded using atomic force microscopy (AFM). Cells were pretreated for 24 h with different combinations of methanol (control), TA, IXB, 2,4-D or IAA using the concentrations indicated in each experiment. A 40 μl-aliquot of each culture was laid on a poly-L-lysine (0.1 mg mL-1) coated slide cover slip for 5 min to allow adhesion. The cover slip was washed three times with culture medium before fixing it with a minute drop of vacuum grease in a small petri dish that was filled with 2 mL of culture medium prior to analysis. AFM analysis was conducted in contact mode as described before [41]. Briefly, AFM studies were performed with a JPK instrument NanoWizard® 4 V.6 (Berlin, Germany) mounted on top of an inverted Zeiss imager Z1 microscope (Carl Zeiss Canada Ltd., Ontario, Canada). MLCT cantilevers A with a nominal spring constant of 0.07 N/m were used (Bruker AFM probes, California, USA). For this cantilever we typically obtain a spring constant ranging from 0.05 to 0.11 N/m, using the thermal noise technique [76]. The Young’s modulus was calculated using the Hertz model adapted for a four-sided pyramid indenter, built-in the JPK analysis software. The Young’s modulus of the glass cover slip was considered infinitely rigid when compared to that of the measured cells. All studies were carried at room temperature. Force-curves were recorded at five different arbitrary locations in a 5 μm radius over the cell surface leading to five batches of 30 to 50 points per cell. We used 3 to 4 cells per experiment. Each experiment was repeated 3 times.

### Statistics

Statistical analysis was performed with GraphPad Prism 7. For cell death assay and measurements, cells were counted in groups of 100 and the mean was calculated from 3 to 15 replicates. Each experiment was repeated at least three times. Data was analyzed using t-test followed by Holm-Šídák method with alpha = 0.05. Results were considered statistically different when *p*-value was < 0.05.

## Supplementary information

**Additional file 1: Figure S1.** Visualization of cell death in *Arabidopsis* suspension-cultured cells 48 h after the addition of cellulose biosynthesis inhibitor (CBI), lanthanum chloride (LaCl_3_), ruthenium red (RR), or CBI with auxin using epifluorescence microscopy (left) or light microscopy (right). Treatments: **a)** Control (methanol); **b)** Thaxtomin A (TA: 1 μM); **c)** Isoxaben (IXB: 1 μM); **d)** LaCl_3_ (500 μM); **e)** RR (50 nM); **f)** TA (1 μM) + 2,4-dichlorophenoxyacetic acid (2,4-D: 50 μM); **g)** IXB (1 μM) + IAA (1 μM). Left panels: Cells were stained with propidium iodine (PI) and fluorescein diacetate (FDA) to detect dead (red) and living cells (green). Right panels: Cells were stained with trypan blue to detect dead cells (black). Images were taken with upright microscope Zeiss Z1 imager. Scale = 100 μm.

**Additional file 2: Figure S2.** Auxin increases cell survival in thaxtomin A-treated cells. Percentage of cell death in *Arabidopsis* suspension-cultured cells at the indicate time after treatment with: 2,4-dichlorophenoxyacetic acid (2,4-D: 50 μM), thaxtomin A (TA: 1 μM), indole-acetic acid (IAA: 30 μM), 1-naphthaleneacetic acid (NAA; 30 μM) or combined treatments of TA with either 2,4-D, IAA or NAA. Each time point represents the average value of three different experiments including 500 cells each. Error bars indicate SD. Statistically different values (t-test followed by Holm-Šídák method, *p* < 0.05) are indicated by a different letter within each time point.

**Additional file 3: Figure S3.** Auxin increases cell survival in isoxaben-treated cells. Percentage of cell death in *Arabidopsis* suspension-cultured cells at the indicate time after treatment with: 2,4-dichlorophenoxyacetic acid (2,4-D: 1 μM), isoxaben (IXB: 1 μM), indole-acetic acid (IAA: 1 μM), 1-naphthaleneacetic acid (NAA; 1 μM) or combined treatments of IXB with either 2,4-D, IAA or NAA. Each time point represents the average value of three different experiments including 500 cells each. Error bars indicate SD. Statistically different values (t-test followed by Holm-Šídák method, *p* < 0.05) are indicated by a different letter within each time point.

**Additional file 4: Table S1.** Thaxtomin A-induced cell death in plasmolysed cells.

## Data Availability

The datasets used and/or analysed during the current study are available from the corresponding author on reasonable request.

## Abbreviations

2,4-D: 2,4-Dichlorophenoxyacetic acid
AFM: Atomic Force Microscopy
CBI: Cellulose Biosynthesis Inhibitor
CESA: Cellulose Synthase
CSC: Cellulose Synthesis Complex
CTRL: Control
IAA: Indole-3-Acetic Acid
IXB: Isoxaben
PCD: Programmed Cell Death
TA: Thaxtomin A
