# Supplementary material for: Auxin protects Arabidopsis thaliana cell suspension cultures from programmed cell death induced by the cellulose biosynthesis inhibitors thaxtomin A and isoxaben
Source: BMC Plant Biol. 2019 Nov 21;19:512. doi: 10.1186/s12870-019-2130-2 (PMC6873746; doi:10.1186/s12870-019-2130-2)
Supplement: Supplementary file 1 — Additional file 1: Figure S1. Visualization of cell death in Arabidopsis suspension-cultured cells 48 h after the addition of cellulose biosynthesis inhibitor (CBI), lanthanum chloride (LaCl3), ruthenium red (RR), or CBI with auxin using epifluorescence microscopy (left) or light microscopy (right). Treatments: a) Control (methanol); b) Thaxtomin A (TA: 1 μM); c) Isoxaben (IXB: 1 μM); d) LaCl3 (500 μM); e) RR (50 nM); f) TA (1 μM) + 2,4-dichlorophenoxyacetic acid (2,4-D: 50 μM); g) IXB (1 μM) + IAA (1 μM). Left panels: Cells were stained with propidium iodine (PI) and fluorescein diacetate (FDA) to detect dead (red) and living cells (green). Right panels: Cells were stained with trypan blue to detect dead cells (black). Images were taken with upright microscope Zeiss Z1 imager. Scale = 100 μm. [file 12870_2019_2130_MOESM1_ESM.pdf]

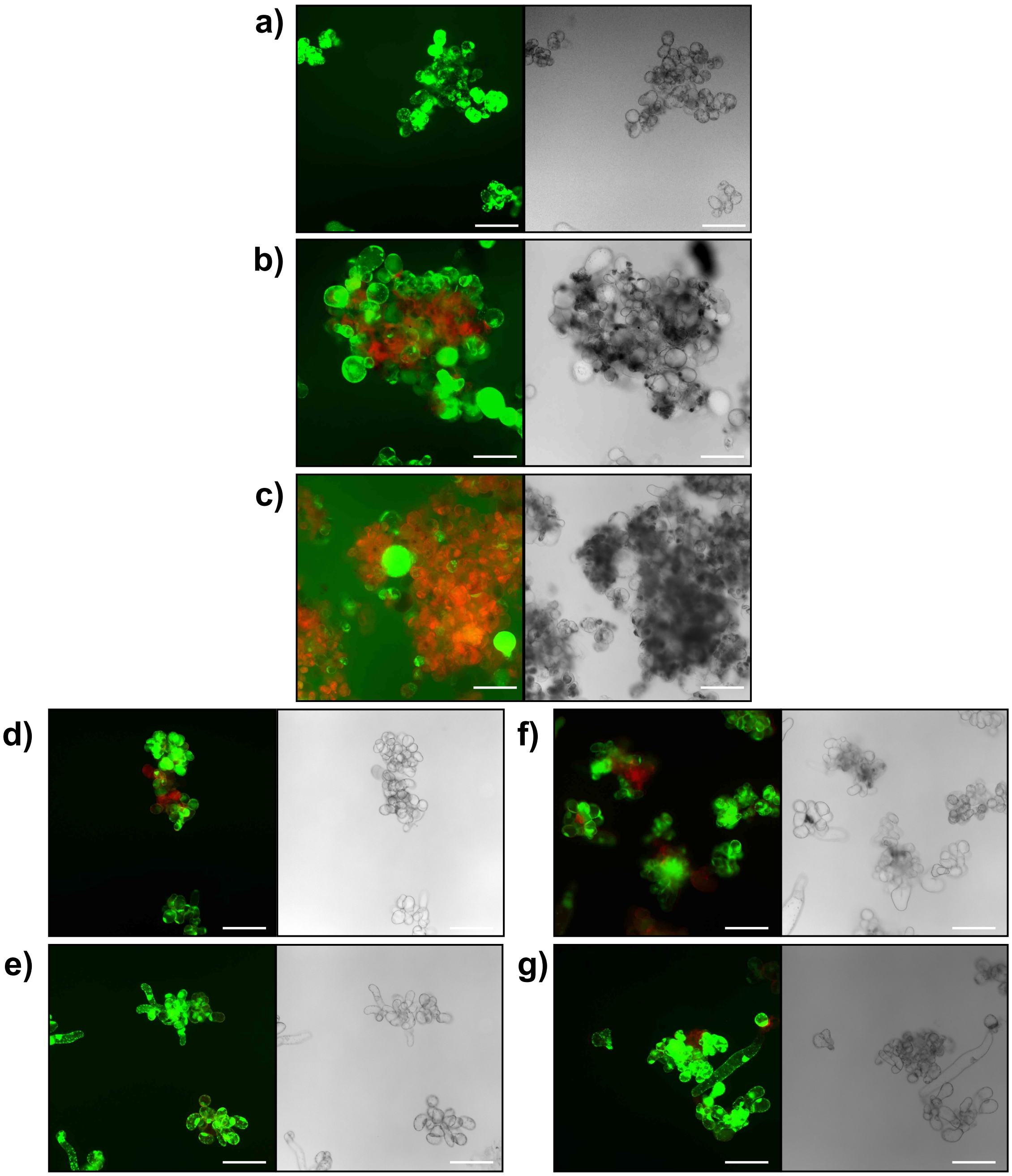

Figure S1. Visualization of cell death in *Arabidopsis* suspension-cultured cells 48 h after the addition of cellulose biosynthesis inhibitor (CBI), lanthanum chloride ($LaCl_3$), ruthenium red (RR), or CBI with auxin using epifluorescence microscopy (left) or light microscopy (right).

Treatments: **a)** Control (methanol); **b)** Thaxtomin A (TA: 1 µM); **c)** Isoxaben (IXB: 1 µM); **d)** $LaCl_3$ (500 µM); **e)** RR (50 nM); **f)** TA (1 µM) + 2,4-dichlorophenoxyacetic acid (2,4-D: 50 µM); **g)** IXB (1 µM) + IAA (1 µM).

Left panels: Cells were stained with propidium iodine (PI) and fluorescein diacetate (FDA) to detect dead (red) and living cells (green). Right panels: Cells were stained with trypan blue to detect dead cells (black). Images were taken with upright microscope Zeiss Z1 imager. Scale = 100 µm.
