# Supplementary material for: Auxin protects Arabidopsis thaliana cell suspension cultures from programmed cell death induced by the cellulose biosynthesis inhibitors thaxtomin A and isoxaben
Source: BMC Plant Biol. 2019 Nov 21;19:512. doi: 10.1186/s12870-019-2130-2 (PMC6873746; doi:10.1186/s12870-019-2130-2)
Supplement: Supplementary file 2 — Additional file 2: Figure S2. Auxin increases cell survival in thaxtomin A-treated cells. Percentage of cell death in Arabidopsis suspension-cultured cells at the indicate time after treatment with: 2,4-dichlorophenoxyacetic acid (2,4-D: 50 μM), thaxtomin A (TA: 1 μM), indole-acetic acid (IAA: 30 μM), 1-naphthaleneacetic acid (NAA; 30 μM) or combined treatments of TA with either 2,4-D, IAA or NAA. Each time point represents the average value of three different experiments including 500 cells each. Error bars indicate SD. Statistically different values (t-test followed by Holm-Šídák method, p < 0.05) are indicated by a different letter within each time point. [file 12870_2019_2130_MOESM2_ESM.pdf]

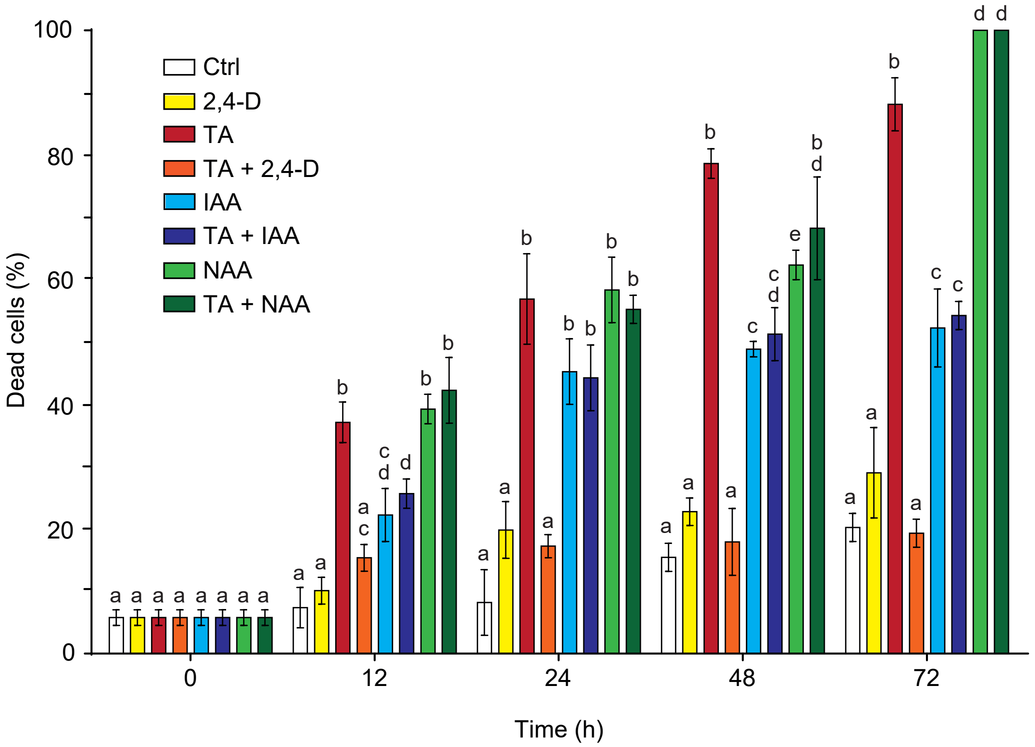

Figure S2. Auxin increases cell survival in thaxtomin A-treated cells.

Percentage of cell death in *Arabidopsis* suspension-cultured cells at the indicate time after treatment with: 2,4-dichlorophenoxyacetic acid (2,4-D: 50 μM), thaxtomin A (TA: 1 μM), indole-acetic acid (IAA: 30 μM), 1-naphthaleneacetic acid (NAA; 30 μM) or combined treatments of TA with either 2,4-D, IAA or NAA.

Each time point represents the average value of three different experiments including 500 cells each. Error bars indicate SD. Statistically different values (t-test followed by Holm-Šídák method, $p < 0.05$) are indicated by a different letter within each time point.
