# Supplementary material for: Auxin protects Arabidopsis thaliana cell suspension cultures from programmed cell death induced by the cellulose biosynthesis inhibitors thaxtomin A and isoxaben
Source: BMC Plant Biol. 2019 Nov 21;19:512. doi: 10.1186/s12870-019-2130-2 (PMC6873746; doi:10.1186/s12870-019-2130-2)
Supplement: Supplementary file 4 — Additional file 4: Table S1. Thaxtomin A-induced cell death in plasmolysed cells. [file 12870_2019_2130_MOESM4_ESM.pdf]

Table S1. Thaxtomin A-induced cell death in plasmolysed cells[1].

Condition	Cell death[2] (%) ± SD
TA (1 µM)	48.25 ± 8.25
Plasmolysed cells + TA (1 µM)	29.45 ± 8.75

[1] *Arabidopsis* cell suspensions were plasmolysed by adding 0.1 M Mannitol for 1 h before adding TA.

[2] Dead cells were counted using trypan blue viability assay 48 h after addition of thaxtomin A (TA) (1 µM). Values represent the mean percentage of cell death ± SD for three different samples per condition. 150 cells were counted per sample.
